# Double Orchectomy for Prostatic Enlargement

**Published:** 1897-02-06

**Authors:** J. Mackenzie Booth

**Affiliations:** Surgeon and Lecturer on Clinical Surgery in the Royal Infirmary, Aberdeen, Lecturer on Diseases of the Ear and Larynx in Aberdeen University.


### DOUBLE ORCHECTOMY FOR PROSTATIC ENLARGEMENT.

By J. MACKENZIE BOOTH, C.M., M.A., M.D., Surgeon and Lecturer on Clinical Surgery in the Royal Infirmary, Aberdeen, Lecturer on Diseases of the Ear and Larynx in Aberdeen University.

During the last few years numerous cases of castration for enlarged prostate have been reported, but the recorded cases are still too few, and the time that has elapsed since the institution of the operation is still too short, for the due estimation of its value. A short notice of the following hitherto unrecorded case, eighteen months after operation, may, therefore, possess sufficient interest to justify its publication.

In the middle of May, 1895, a professional friend in one of our rural practices wrote to me requesting me to admit into the hospital for treatment a patient of his, living in a remote part of his district, who suffered from considerable enlargement of the prostate. He said that he had also had repeated attacks of inflammation of the testes, and often had retention of urine, requiring the use of the catheter, which was very difficult, adding a suggestion of the probable benefit of operative interference.

On May 29th the patient, a tall, strong-looking crofter, sixty-nine years of age, was admitted into the Aberdeen Royal Infirmary. He stated that, more than ten years before, he began to experience difficulty in passing water, having to stand for a minute or two before he could get it, and that the difficulty was always greatest during the night. For several years at first there was but little and a very gradual increase in the symptoms, but for the last three years they had been more aggravated, as he had to walk about for a long time before the urine could be voided, and occasionally required the doctor to catheterise him—always a matter of considerable difficulty. He had also on several occasions suffered from painful attacks of swelling of the testicles, sometimes on the right and sometimes on the left side. As a rule he required to pass water every two hours, but at times much more frequently, and always with much pain. He complained that the bowels were very constipated, and that this greatly aggravated his urinary affection. Physical examination showed the patient to be fairly well nourished and powerful for his time of life. The respiratory, circulatory, and digestive organs were healthy. Investigation showed some fulness of the hypogastric region, and this was confirmed by palpation and percussion. The testes were large, uneven, almost nodulated, and unduly pendulous—the right being somewhat larger and hanging lower than the left. On digital examination of the prostate per rectum, the gland was sensitive to touch and generally much enlarged, the right lobe being slightly larger than the left. Its substance was seemingly of normal consistence, it was from two to three inches in breadth, while it bulged into the rectum so as partially to obstruct it, and its upper border could not be reached by the examining finger. On being asked to micturate the patient was able to pass about two ounces of urine, the stream being very weak. With some difficulty the smallest available silver prostatic catheter, a No. 9, was passed into the bladder, when 21 ounces of residual urine were withdrawn after nine inches of the catheter had passed into the urethra. The average quantity of urine passed in 24 hours was 47 ounces, turbid, straw-coloured, with a faintly acid reaction, a specific gravity of 1022, a trace of albumen and blood, no sugar, and a copious deposit of pus, mucus, epithelium, and oxalatis.

On consideration of the circumstances of the case, *i.e.*, the painful micturition, the occasional retention, the difficulty of catheterisation, the repeated attacks of orchitis, and the remoteness from medical aid, double castration was recommended, and the patient gave a ready assent.

On June 5th, after disinfection of the parts, both testes were removed by a vertical incision over the upper part of each, the vessels were secured by double ligatures, small drains were inserted, and the wounds were closed by continuous sutures and dressed with double cyanide gauze.

There was considerable pain and desire to pass water after the operation, and slight hæmaturia, which ontinued more or less for ten days, the catheter being passed twice or thrice daily, and the residual urine varying from 17 to 20 ounces. The wounds were dressed on the second day, and after that daily till June 20th, when they were healed.

On the twelfth day after the operation the symptoms began to show distinct signs of amelioration. The pain grew less, the patient began to pass water freely, the blood disappeared from the urine, and the prostate per rectum felt smaller, flatter, and puckered.

The length of urethra traversed by the catheter before urine began to flow also got less, so that when the patient was last catheterised on June 30th scarcely, eight inches were passed. The urine became almost normal, the pus, albumen, and blood disappearing, and only a little excess of mucus was noted. The quantity of residual urine was much diminished, and the patient only required to rise twice or thrice during the night. Once during the 14th and again on the 18th of June there was a slight rigor and rise of temperature to 99·8 degrees and 100·2 degrees respectively, for which two grains of sulphate of quinine were given twice daily. On June 23rd a small trocar was passed into the left side of the scrotum and half an ounce of bloody serum was removed. Otherwise recovery was rapid and complete. The patient was dismissed on July 1st, when he said he felt no distress, and passed urine without any difficulty.

On November 22nd, 1896, I received the following statement from his medical attendant: "The patient has not required to have a catheter passed since he left the hospital eighteen months ago. The frequency of micturition is still present, the patient requiring to pass water every two hours or so, but there is no pain, and scarcely any difficulty. Occasionally he has to stand half a minute or so before getting a start, but the urine comes freely once it begins, and he has no dribbling at any time. His urine has been for long perfectly clear and free from pus. Per rectum, his prostate is certainly large and pretty firm—about two inches in vertical and transverse diameter—but I believe it is flattened antero-posteriorly, as it does not bulge at all into the rectum. I meant to pass a catheter after urination to test him for residual urine, but his urine was so clear and right that I feared the risk of setting up fresh trouble. He is in robust health, working hard, and having an occasional bout when he goes from home —a thing he always liked to do, but which for several years was invariably followed by an attack of retention."

The foregoing case illustrates well the value of operative interference in otherwise hopeless cases of prostatic enlargement. The painful micturition, the attacks of retention, the difficulty of catheterisation, the repeated occurrence of orchitis, and the remoteness of the patient from medical aid, rendered his condition an exceptionally distressing one, and urgently demanded some measure giving permanent relief. In less than a month after operation the patient had exchanged a condition of misery for one of comparative comfort, which has been maintained up till now, in spite of a tendency to occasional excess in the use of alcohol. The rapid change in the condition of the urine was also very striking. In little more than a fortnight, the turbid, mucopurulent, often bloody, urine of cystitis and prostatic engorgement had given place to a clear, bland, and almost normal secretion. The contractile power of the bladder, too, had evidently become much greater while in hospital, though I have been unable to ascertain the presence or amount of residual urine at this time.

Regarding the selection of operation, the unhealthy states of the testes pointed to double castration as the procedure most likely to be useful in this case. The more recently introduced method of resection of one or both vasa (vasectomy), which will undoubtedly to some extent supersede castration, or the removal of one testis, would have left behind an unhealthy organ, and probably given tardy or insufficient relief; while perineal section or prostatectomy would have involved a greater risk, without any corresponding advantage.

It is difficult to say with exactitude to what extent the prostate has diminished, but that it has done so considerably admits of no reasonable doubt. More especially was this manifest in its length and thickness as ascertained by catheterisation and rectal examination, while the apparent breadth had not so evidently been reduced.

Of the mental disturbance that has been noted in some cases after castration there has been no indication. On the contrary, his mental as well as his bodily health has been improved, and his prospect of longevity considerably enhanced.

